# Early Maladaptive Schemas and Muscle Dysmorphic Symptoms in Adolescents

**DOI:** 10.1007/s40653-026-00894-3

**Published:** 2026-05-02

**Authors:** Shanyan Lin, Claudio Longobardi, Matteo Angelo Fabris

**Affiliations:** Department of Psychology, University of Turin, Via Verdi 10, 10124 TO Turin, Italy

**Keywords:** Early maladaptive schemas, Adolescents, Muscle dysmorphia, Gender

## Abstract

The aim of the study is to investigate the possible association between early maladaptive schemas (EMS), emanating from traumatic relationships with attachment figures and acknowledged as a risk factor that increases the susceptibility to various forms of psychopathology, notably muscle dysmorphia (MD) within a cohort of Italian adolescents. The sample consisted of 1793 Italian high school students aged 13–20 years (*M*_*age*_ = 16.69, *SD* = 1.66; 59.3% female). Adolescents completed an anonymous questionnaire containing measures of EMS and MD, as well as sociodemographic data. Our data show that MD is significantly and positively correlated with total EMS scores and individual schemas, except for Enmeshment, with which there is a significant but negative correlation. There are no significant gender differences in the correlation between MD and EMS (total scores and individual schemas). In addition, regression analyses show that male gender and the Disconnection/Rejection, Other-directedness and Overvigilance/Inhibition tended to predict more MD symptoms, whereas the Impared Limits domain was negatively associated with MD. The findings support a possible association between MD and specific EMS, consistent with the cognitive theory of psychopathology, which states that each disorder is associated with specific cognitive vulnerabilities. These findings underscore the role of trauma-related relational experiences and schema-based vulnerabilities in the development of MD during adolescence, highlighting the importance of early identification and schema-focused prevention and intervention approaches. Future longitudinal research is needed to clarify developmental trajectories and the mechanisms linking early adverse experiences, EMS, and MD symptomatology.

## Introduction

### Muscle Dysmorphia in Adolescence

Adolescence is a critical period for identity development and body image becomes a prominent element of adolescents' self-concept (Fabris & Longobardi, 2024). Adolescents are particularly vulnerable to developing body dissatisfaction (Longobardi et al., 2021; Myers & Crowther, 2009), which is a risk factor for negative developmental outcomes, including mental health disorders such as body dysmorphic disorder (BDD) (Karazsia et al., 2017). BDD is a severe psychopathological disorder characterized by excessive and irrational concerns about perceived flaws of one's physical appearance that are in fact unnoticed by others or are definitely negligible (APA, 2013; Fabris et al., 2022). With the publication of the DSM-5 (APA, 2013), the scientific community formally recognized a specific subtype of BDD, namely muscle dysmorphic disorder (MD). Individuals suffering from MD exhibit dysmorphic concerns about their muscles, which they perceive as not sufficiently developed or lean enough (Pope et al., 1997, 2005). This condition can cause significant distress and limit the individual's social functioning. Individuals with high levels of MD may resort to social avoidance strategies, driven by the shame and anxiety stemming from their physical appearance (Martenstyn et al., 2022; Olivardia et al., 2000). Additionally, they engage in risky and unhealthy behaviors, such as excessive exercise, inappropriate dieting, and the use of anabolic substances to enhance their musculature (Badenes-Ribera et al., 2019a, b; Pope et al., 2005; Settanni et al., 2018). In general, several studies seem to support an association between MD and a variety of psychological symptoms in both adults (Dèttore et al., 2020; Longobardi et al., 2017; Mitchel et al., 2017) and adolescents (Ganson et al., 2023), indicating the clinical complexity of the syndrome and the associated risks for psychological adjustment and development of affected individuals.

Although MD is considered a typically male disorder, likely due to the association between masculinity and muscularity in Western cultures (Badenes-Ribera et al., 2018; 2021; Fabris et al., 2018; Ganson et al., 2023), females are not immune to it (Hale et al., 2013; Robert et al., 2009). The prevalence of MD, particularly in adolescence, is uncertain, but recent evidence suggests that approximately 2.2% of males and 1.4% of females have been diagnosed with MD (Mitchison et al., 2022). Generally, the literature appears to primarily focus on adults and individuals engaged in sports (e.g., bodybuilding, Rubio-Aparicio et al., 2020), whereas only more recently have community samples of adolescents been considered. This is important because MD typically emerges during late adolescence (Olivardia, 2001) and positive psychological adjustment during adolescence may serve as a protective factor against adverse developmental outcomes in adulthood.

### Early Maladaptive Schemas and Psychological Adjustment in Adolescence

Although the risk factors associated with MD have been widely explored, further research is needed to better understand the etiology of the syndrome. Recent studies (e.g., Longobardi et al., 2022) suggest the importance of considering traumatic or adverse childhood experiences in the study of body dysmorphic disorder. Along these lines, *early maladaptive schemas* (EMS), originating from traumatic relationships with attachment figures and recognized as risk factor for various forms of psychopathology, may increase vulnerability to the development of MD during adolescence. EMS are conceptualized as a broad, pervasive, and dysfunctional schemas that include cognitive, affective, memory, and physical sensations regarding oneself and one's relationships with others (Young et al., 2006). EMS originate in early childhood and develop from complex interactions between the child's temperament and a relational context characterised by traumatic events and constant primary care giving relationships in which core emotional needs (such as the need for safety and emotional support) are not met (Bach et al., 2018; Pilkiltong et al., 2021). Prolonged exposure to a negative, neglectful, or abusive parental relationship can lead to the development of a particularly rigid and complex set of negative beliefs about the self, the world, and the future (schemas) that influence how the individual interprets events and interpersonal relationships (Beck & Young, 1985). While originating in early childhood, EMS tend to be revised during adolescence and adulthood in response to experiences in relationships with significant others. Young et al. (2006) identified 18 EMS (see Appendix), divided into five general domains, each characterized by specific unmet emotional needs. The five domains are: (1) *Disconnection and Rejection*: the needs for secure attachment to others and thus for acceptance, closeness, and caring are not met by caregivers. Schemas in this domain are usually related to perceptions of self as fragile and insecure and of others as abandonment-prone, abusive, and emotionally unavailable; (2) *Impaired Authonomy and Performance*: the need for autonomy, competence, and sense of identity are the unmet emotional needs in the relationship with caregivers. Schemas in this area are related to perceptions of oneself as immature, incompetent, and unable to achieve on one's own. Caregivers tend to be overprotecting and family members tend to become enmeshed; (3) *Impaired Limits*: This area is characterized by schemas related to the lack of limits and self-control and the belief that they are superior to others. Parenting in these relationships is often characterized by a lack of discipline, overindulgence, or permissiveness; (4) *Other-directedness*: the need to express one's feelings and needs freely and spontaneously is not met. Schemas focus almost exclusively on the desires and feelings of others in order to gain approval. These are usually children who perceive the needs of caregivers as more important than their own and experience conditional acceptance in their relationship with their parents; (5) *Over-vigilance and Inhibition*: lack of spontaneity, relaxation, and playfulness. These schemas are related to the need to follow rigid rules and high standards of perfection and to suppress the expression of one's needs, thoughts, and feelings in order to avoid negative consequences. Usually, caregivers tend to be pessimistic and punitive.

EMS act as lenses that help the subject make sense of reality, interpret events, organise information, and guide thought and behaviour. Most people possess EMS that remain inactive until they are trigged by a stressor or challenging situation, which can increase the risk of developing psychological disorders and relationship difficulties (Tariq et al., 2021b). In this sense, EMS are considered as strong cognitive vulnerabilities associated with various forms of psychological disorders. Several studies support the role of EMS in the risk of developing various forms of mental disorders in adolescents and young adults (Nicol et al., 2020; Van Vlierberghe et al., 2010), but no study has examined a possible link between EMS and MD. This is a limitation considering the potential negative impact of MD on adolescents' psychological adjustments and social functioning. Investigating the possible association between MD and EMS can contribute to our understanding of the possible mechanisms underlying the syndrome, detecting cognitive vulnerabilities that may increase the risk of developing MD, and the implementation of the most appropriate prevention and treatment strategies.

### Early Maladaptive Schemas and Muscle Dysmorphia: a Possibile Link?

EMS can be considered a transdiagnostic factor for mental disorders, although several studies indicate that specific EMS are more likely than others to be associated with specific psychopathology (Bar et al., 2023). Accordingly, it is important to investigate EMS in the context of less studied conditions, such as MD. In addition, it would allow us to explore the frustrated emotional needs and cognitive processes that may promote or maintain the syndrome and thus develop appropriate assessment, intervention, and prevention strategies.

To date, no study has directly examined the relationship between EMS and MD symptoms in adolescence. However, emerging evidence suggests the plausibility of such a link. First, empirical research has found an association between EMS and a number of psychological symptoms that are considered strongly associated with MD in the literature, such as eating disorders (Maher et al., 2022; Nicol et al., 2020), social anxiety (Calvete, 2014; Calvete et al., 2013; Tariq et al., 2021a, b), depressive symptoms (Tariq et al., 2021b), obsessive–compulsive disorder (Dostal & Pilkington, 2023), suicidal ideation (Pilkington et al., 2021a, b), and exercise dependence (Rankin et al., 2021). In addition, Fabris et al. (2018, 2021) were the first to demonstrate a relationship between MD and frustrated needs for affection, protection, and acceptance in relationships with caregivers from an attachment theory perspective (Bowlby, 1973). Specifically, the authors (Fabris et al., 2018) found that male bodybuilders with high MD scores tended to report a more negative view of others and interpersonal relationships, displayed avoidance strategies of intimate relationships, and attempted to demonstrate an invulnerable, self-sufficient self. According to the authors, these negative representations of self and relationships stemmed from previous relationship experiences with caregivers that were characterised by indifference, rejection, or criticism and were therefore internalised, by defining the representation of the self as a fragile and unlovable person and the other as a person prone to abandonment and rejection. Similarly, Badenes-Ribera et al. (2021) found an association between parental criticism and MD symptoms in a sample of adolescents of both genders and identified perceptions of alienation from parents as a mediating factor. In this sense, criticism would undermine emotional needs for protection, acceptance, and nurturance which would lead to perceiving the relationship with parents as negative, thus internalising negative perceptions of self and others that could produce distorted cognitions. Some research also reports a correlation between MD symptoms and peer victimisation (Fabris et al., 2020, 2021; Wolke & Sapouna, 2008). Importantly, although EMS are structured in the context of early interactions with caregivers, they may be redefined in relation to experiences with significant others during adolescence. And it is during adolescence that peer relationships become a primary source of intimacy and nurturance (Badenes-Ribera et al., 2019a, b), and in this way, peer victimisation may contribute to the development of EMS at this developmental stage. In particular, Fabris et al. (2021) have provided evidence for the mediating role of peer attachment in the relationship between peer victimisation and MD risk in adolescence. Overall, therefore, Fabris et al. (2018, 2020, 2021, 2022) propose that MD can be viewed as an extreme attempt by individuals to compensate for feelings of vulnerability and fears of abandonment, rejection, and judgment, which are likely to result from prior dysfunctional and traumatic experiences with significant reference figures (e.g., parents and peers). According to the authors, individuals may attempt to build a type of armor through musculature that protects them from negative feelings resulting from self-perceptions and expectations in interpersonal relationships. This emotional armor is developed in the body to prevent against judgment, criticism, and abandonment, and to project an image of themselves to others as autonomous, independent, and invulnerable. These interpretations sound fascinating, but they require additional empirical investigation. In this regard, EMS could contribute, but as far as our knowledge extends, no study has undertaken an investigation into the potential relationship between MD symptoms and EMS.

### The Aim of the Study

Based on the above, the aim of our study is to extend current knowledge on factors involved in the etiology of MD in adolescence. Specifically, in this study, we aim to examine the association between EMS and MD symptoms in a sample of adolescents. However, we hypothesize that high levels of MD will be associated with higher levels of EMS. We also want to observe whether and which EMS subtypes are associated with increased MD symptoms. Finally, we investigate whether gender moderates the association between EMS (total and subtypes) and MD symptoms.

## Method

### Participants

Participants comprised of 1787 high school students, aging from 13 to 20 years old (*M* = 16.69, *SD* = 1.66). Of these, 1060 female students (59.3%). Participants were enrolled in five different grades in Italian high schools: Grade 9 (*n* = 338, 18.9%), Grade 10 (*n* = 333, 18.6%), Grade 11 (*n* = 206, 11.5%), Grade 12 (*n* = 311, 17.4%), Grade 13 (*n* = 583, 32.6%). A total of 16 students with missing grade information (0.9%). The majority of the participants (61.9%, *n* = 1106) were engaged in sports, and 41.3% (*n* = 738) reported regularly attending a gym.

### Instruments

#### Socio-demographic Variables

Participants completed an anonymous questionnaire containing a section of socio-demographic data that included: age, gender, engaged in a sport (yes/no), and going to the gym (yes/no).

#### Early Maladaptive Schemas

To measure EMS, we used the Brief form of the Young Schema Questionnaire for Adolescents (B-YSQ-A, Santos et al., 2018). This instrument consists of 54 self-reported items measuring 18 subtypes of EMSs (e.g., social isolation/alienation, emotional inhibition). Each EMSs subtype is measured by 3 items. Participants were asked to rate the items using a six-point scale from 1 (*It has nothing to do with me*) to 6 (*It is exactly what happens to me*). In the current sample, the reliability of EMS was good (Cronbach’s alpha = 0.93), and the reliabilities of each subscale were also satisfactory, with Cronbach’s alphas ranging from 0.61 to 0.89.

#### Muscle Dysmorphic Symptoms

MD symptoms were assessed using the Muscle Dysmorphic Disorder Inventory (MDDI, Hildebrandt, Langenbucher, & Schlundt, 2004; Italian version of Santarnecchi & Dèttore, 2012). The MDDI is a multi-dimensional self-report measure to measure the risk of muscle dysmorphia. It is composed of 13 items structured around three dimensions: Desire for Size (5 items), Appearance Intolerance (4 items), and Functional Impairment (4 items). For the purposes of this study, total scores were calculated, which is the sum of the scores obtained on the individual subscales and reflects the general level of MD symptoms. High levels indicate an increased risk of the subject reporting MD symptoms. In the current sample, the reliability of MDDI was acceptable (Cronbach’s alpha = 0.68).

### Procedure and Ethical Considerations

The study was conducted in several high schools in northern and central Italy. In total, 7 schools were contacted, and only 4 agreed to take part in the research. After approval from school principals, the students were invited to participate in the study, which involved filling in an anonymous questionnaire. It was explained to them that participation in the research was free and voluntary, without compensation. For underage students, parental permission was obtained. The study was approved by the ethics committee of the Institutional board (N. 0318535) and was conducted in accordance with the guidelines of the Italian Association of Psychology (AIP) and current privacy regulations.

### Strategy of Analysis

Data were analyzed using SPSS 29.0. First, descriptive and correlational analyses were conducted on the full sample, as well as in female and male subsamples separately. Gender differences in the subtypes of EMS, as well as on the correlations between EMS and MD symptoms, were explored. Second, participants were divided into a high MD risk group (MDDI score > 39) and a low MD risk group (MDDI score ≤ 39) according to cut-off criteria used in previous studies (e.g., Fabris et al., 2018, proposed by Varangis et al., 2012) and group differences in EMS were examined. Third, regression analyses were conducted to further explore the relationship between the five domains of EMS and the MD symptoms.

## Results

### Descriptive Statistics and Correlations

The means and standard deviations of overall EMS and its 18 subtypes are presented in Table 1 for the full sample, as well as for the female and male subsamples. Generally, gender differences were observed in both overall EMS scores and most subtype levels. Specifically, female students’ overall EMS (*M* = 3.06, *SD* = 0.68) was significantly higher than that of male students (*M* = 2.78, *SD* = 0.66) significantly (*t* = 8.64, *p* < .001). Among the 18 EMS subtypes, four subtypes (i.e., Dependence/ incompetence, Entitlement/ grandiosity, Subjugation, Punitiveness) did not differ significantly between female and male students. Female students had higher scores on most of the 18 EMS subtypes, whereas male students had higher scores on Enmeshment/ undeveloped self (*t* = –4.65, *p* < .001) and Unrelenting standards/ hyper-criticalness (*t* = –2.19, *p* = .029).

**Table 1:** Means and standard deviations of EMS in full sample and subsample by genders

EMS	M (SD)			t	*p*
	Full sample (*N* = 1787)	Female subsample (*n* = 1060)	Male subsample (*n* = 704)		
Abandonment/ instability	4.12 (1.38)	4.46 (1.29)	3.60 (1.37)	13.45***	< .001
Mistrust/ abuse	3.68 (1.24)	3.88 (1.21)	3.37 (1.20)	8.64***	< .001
Emotional deprivation	2.15 (1.19)	2.21 (1.20)	2.03 (1.13)	3.14**	.002
Defectiveness/ shame	2.70 (1.44)	2.97 (1.49)	2.25 (1.22)	10.6***	< .001
Social isolation/alienation	2.91 (1.43)	3.05 (1.44)	2.67(1.37)	5.60***	< .001
Dependence/ incompetence	2.17 (1.00)	2.17(0.99)	2.13(0.99)	0.88	.381
Vulnerability to harm or illness	2.64 (1.21)	2.82(1.22)	2.35(1.11)	8.17***	< .001
Enmeshment/ undeveloped self	2.78 (1.34)	2.67(1.37)	2.97(1.24)	–4.65***	< .001
Failure	2.61 (1.39)	2.74(1.44)	2.37(1.26)	5.55***	< .001
Entitlement/ grandiosity	2.40 (1.07)	2.38(1.05)	2.41(1.09)	–0.49	.627
Insufficient self-control and/or self-discipline	2.71 (1.18)	2.74(1.19)	2.62(1.11)	2.25*	.025
Subjugation	2.30 (0.99)	2.32(1.01)	2.26(0.95)	1.22	.221
Self-sacrifice	3.88 (1.25)	4.14(1.25)	3.5(1.15)	10.9***	< .001
Approval-seeking/ recognition seeking	3.19 (1.19)	3.29(1.21)	3.01(1.12)	4.82***	< .001
Negativity/ pessimism	3.47 (1.42)	3.67(1.43)	3.14(1.35)	7.75***	< .001
Emotional inhibition	3.27 (1.31)	3.46(1.31)	2.97(1.25)	7.79***	< .001
Unrelenting standards/ hyper-criticalness	3.27 (1.35)	3.2(1.36)	3.35(1.34)	–2.19*	.029
Punitiveness	2.99 (1.16)	2.94(1.17)	3.04(1.13)	–1.80	.071
Total EMS	2.96 (0.69)	3.06(0.68)	2.78(0.66)	8.64***	< .001

*EMS* Early Maladaptive Schemas; there were 23 students did not provide their gender information; *p < .05, **p < .01, ***p < .001

Table 2 shows the correlations between EMS and MD both in the full sample and in gender-specific subsamples. The overall EMS was positively correlated with students’ MD symptoms in the full sample (*r* = 0.45, *p* < .001), as well as in female subsample (*r* = 0.46, *p* < .001) and male subsample (*r* = 0.42, *p* < .001). Similarly, significant positive correlations between most EMS subtypes and MD symptoms were observed, with the exception of the correlation between Enmeshment/ undeveloped Self and MD symptoms. Specifically, Enmeshment/ undeveloped Self was negatively associated with MD symptoms in both the overall sample and gender-specific subsamples. Subsequently, Fisher's *z* transformation (Fisher, 1915) was applied to convert the correlation coefficients, allowing z-tests to be conducted to compare the correlation coefficients between female and male students. The results revealed no significant gender differences in the correlation coefficients between EMS and MD.

**Table 2:** Correlations between EMS and MD in full sample and subsample by genders

EMS	Correlations with MD			z	*p*
	Full sample (*N* = 1787)	Female subsample (*n* = 1060)	Male subsample (*n* = 704)		
Abandonment/ instability	.32***	.31***	.31***	0.00	1.00
Mistrust/ abuse	.31***	.29***	.32***	0.68	.497
Emotional deprivation	.32***	.31***	.33***	0.46	.647
Defectiveness/ shame	.40***	.42***	.36***	1.45	.146
Social isolation/alienation	.32***	.32***	.30***	0.45	.650
Dependence/ incompetence	.25***	.25***	.22***	0.65	.514
Vulnerability to harm or illness	.33***	.33***	.31***	0.46	.647
Enmeshment/ undeveloped self	–.22***	–.17***	–.26***	1.94	.053
Failure	.25***	.25***	.24***	0.22	.827
Entitlement/ grandiosity	.11***	.09**	.14***	1.04	.298
Insufficient self-control and/or self-discipline	.18***	.15***	.21***	1.27	.203
Subjugation	.22***	.25***	.19***	1.30	.195
Self-sacrifice	.22***	.24***	.18***	1.29	.197
Approval-seeking/ recognition seeking	.31***	.27***	.34***	1.59	.113
Negativity/ pessimism	.35***	.37***	.31***	1.39	.163
Emotional inhibition	.30***	.28***	.30***	0.45	.654
Unrelenting standards/ hyper-criticalness	.18***	.16***	.21***	1.06	.288
Punitiveness	.22***	.26***	.18***	1.73	.084
Total EMS	.45***	.46***	.42***	1.02	.308

*EMS* Early Maladaptive Schemas; *MD* Muscle Dysmorphia; there were 23 participants did not report their gender information. *p < .05, **p < .01, ***p < .001

### The differences between Low and High MD Risk Group and the Regression Results

The results of the EMS differences between the low and high MD risk groups are presented in Table 3. The low MD risk group had lower levels of overall EMS (*M* = 2.93, *SD* = 0.67) than the high MD risk group (*M* = 3.70, *SD* = 0.63) with a large effect size (*t* = –9.51, *p* < .001, Cohen’s *d* = –1.14). Most of the EMS subtypes were lower in low MD risk group with the effect sizes ranging from small to large. However, for the EMS subtype of Enmeshment/ undeveloped Self, the low MD risk group showed higher values (*M* = 2.81, *SD* = 1.33) than high MD risk group (*M* = 2.10, *SD* = 1.18) with a medium effect size (Cohen’s *d* = 0.53). In addition, regression analyses were conducted to further explore the relationship between the five EMS domains and MD symptoms. As shown in Table 4, students with higher values in the EMS domains of Disconnection and rejection (β = 0.29, *p* < .001), Other-directedness (β = 0.11, *p* < .001), and Overvigilance and inhibition (β = 0.16, *p* < .001) tended to have more MD symptoms, whereas EMS in the domain of Impaired autonomy and performance did not significantly predict students’ MD symptoms (β = 0.01, *p* = 0.637). Furthermore, EMS in the Impaired Limits domain negatively predicted MD symptoms (β = –0.07, *p* < .01).

**Table 3:** Means and standard deviations of EMS in low MD risk group and high MD risk group

EMS	M (SD)		t	*p*	Cohen’s d
	Low MD risk group	High MD risk group			
Abandonment/ instability	4.09 (1.38)	4.95 (1.29)	–5.24	< .001	–0.63
Mistrust/ abuse	3.64 (1.23)	4.63 (0.90)	–9.00	< .001	–0.81
Emotional deprivation	2.10 (1.16)	3.28 (1.39)	–7.09	< .001	–1.01
Defectiveness/ shame	2.64 (1.41)	4.11 (1.49)	–8.70	< .001	–1.05
Social isolation/alienation	2.87 (1.41)	4.09 (1.45)	–7.20	< .001	–0.87
Dependence/ incompetence	2.13 (0.97)	2.93 (1.23)	–5.42	< .001	–0.81
Vulnerability to harm or illness	2.60 (1.18)	3.61 (1.40)	–6.02	< .001	–0.85
Enmeshment/ undeveloped self	2.81 (1.33)	2.10 (1.18)	4.40	< .001	0.53
Failure	2.57 (1.37)	3.54 (1.57)	–5.15	< .001	–0.70
Entitlement/ grandiosity	2.39 (1.06)	2.65 (1.18)	–2.08	.038	–0.25
Insufficient self-control and/or self-discipline	2.69 (1.18)	3.18 (1.25)	–3.40	< .001	–0.41
Subjugation	2.28 (0.97)	2.82 (1.23)	–3.68	< .001	–0.55
Self-sacrifice	3.85 (1.24)	4.45 (1.36)	–3.98	< .001	–0.48
Approval-seeking/ recognition seeking	3.16 (1.18)	3.90 (1.23)	–5.21	< .001	–0.63
Negativity/ pessimism	3.43 (1.41)	4.60 (1.22)	–6.93	< .001	–0.83
Emotional inhibition	3.24 (1.31)	4.13 (1.17)	–5.67	< .001	–0.68
Unrelenting standards/ hyper-criticalness	3.24 (1.34)	3.99 (1.44)	–4.63	< .001	–0.56
Punitiveness	2.96 (1.15)	3.56 (1.24)	–4.32	< .001	–0.52
Total EMS	2.93 (0.67)	3.70 (0.63)	–9.51	< .001	–1.14

*EMS*  Early Maladaptive Schemas; *MD* Muscle Dysmorphia

**Table 4:** The effects of five early maladaptive schemas domains on muscle dysmorphia

Predictors	DV: Muscle dysmorphia			
	β	SE	*t*	95% CI for β
Age	0.04	0.02	1.80	[–0.003, 0.081]
Gender	0.05*	0.02	2.02	[0.001, 0.088]
EMS_Disconnection and rejection	0.29***	0.03	8.59	[0.227, 0.362]
EMS_Impaired autonomy and performance	0.01	0.03	0.47	[–0.038, 0.062]
EMS_Impaired limits	–0.07**	0.03	–2.66	[–0.114, –0.017]
EMS_Other-directedness	0.11***	0.03	3.68	[0.051, 0.168]
EMS_Overvigilance and inhibition	0.16***	0.03	5.45	[0.103, 0.220]

*DV * Dependent variable; Gender was coded as 0 = female, 1 = male; *p < .05, **p < .01, ***p < .001

## Discussion

The aim of our study was to extend our knowledge of the possible risk factors of MD in adolescence, focusing on the association between EMS (specific schemas and domains) and MD symptoms. In this direction, our data suggest a significant association between most EMS and MD, indicating that EMS may function as cognitive vulnerabilities involved in the etiology or maintenance of the syndrome. These data appear to be consistent with the international literature suggesting that EMS play a role in promoting mental health problems in adolescents (Nicol et al., 2020). However, to date, no study has examined the relationship between EMS and MD symptoms, so this study represents a new contribution to the current literature. Looking at the specific EMS, it appears that MD symptoms are positively and significantly associated with increased overall levels (total score) of maladaptive schemas and specific EMS (with the exception of the Enmeshment schema, which is significantly but negatively associated). These associations seem to reflect the high level of psychological distress that MD imposes on individuals. In addition, the fact that MD is positively associated with each EMS (with the exception of Enmeshment) may suggest that various maladaptive beliefs underlie the disorder (for simplicity and to avoid repetition, we will address this point by discussing the 5 domains of maladaptive schemas), or it may be due to the high comorbidity that MD has with other psychological disorders (Cooper et al., 2020; Dèttore et al., 2020; Longobardi et al., 2017). Interestingly, the only EMS that significantly but negatively correlates with MD is the Enmeshment Schema. This schema reflects seeking intimacy and emotional engagement with one or more individuals to the point where one's individual identity becomes compromised (Young et al., 2006). The Enmeshment schema is strongly associated with childhood experiences characterized by an overprotective parenting style (Bruyster & Pilkington, 2023). In this sense, parents would have fostered the child's dependence on parental caregivers, thereby suppressing the need for autonomy and independence, which would have fostered the emergence of this schema. However, the relational experiences with the MD subjects' caregivers do not seem to fit this picture. Rather, the few studies that have been conducted seem to indicate the presence of a parental relationship characterized by neglect, disinterest, rejection, or excessive criticism, which could lead MD at-risk individuals to build mental representations characterized by the avoidance of intimate relationships and the demonstration of compulsive independence from the other (Badenes-Ribera et al., 2021; Fabris et al., 2018). However, given that enmeshment and disengagement are two opposite poles on the continuum of the degree of family cohesion (Olson, 2000), this may explain the negative relationship observed between MD and the enmeshment pattern. In addition, we found that high-risk MD adolescents not only had significantly lower levels of Enmeshment, but also showed no significant differences in the Grandiosity/Entitlement schema compared with low-risk MD adolescents. This schema is based on an individual's belief that they are superior to others or is not bound by the rules of reciprocity that apply to normal social interactions (Young et al., 2006). This schema is thought to play a central role in the development of narcissistic personality disorder (Young et al., 2006); however, it appears to reflect the characteristics of grandiose narcissism rather than vulnerable narcissism. Whereas grandiose narcissism is indicative of grandiosity, aggression, and dominance and is associated with parental strategies of overvaluation, vulnerable narcissism reflects undermined self-confidence, perceived inadequacy, and negative affect and tends to be associated with parental relationships characterised by neglect, disinterest, and rejection (Boulter et al., 2023). The characteristics of vulnerable narcissism and associated parental experiences appear to be more compatible with those experienced by individuals with high levels of MD, as presented to us in the literature to date (Fabris et al., 2018). Although the relationship between MD and narcissism is controversial (Dèttore et al., 2020), there are several lines of evidence that MD is associated with vulnerable narcissism rather than grandiose narcissism (Boulter et al., 2023; Boulter & Sandgre, 2022).

Linear regression analyses show a significant and positive association between MD and three domains (*Disconnection/Rejection; Other-Directedness; Overvigilance-Inhibition*) and a significant and negative association with the *Impaired Limits* domain. According to cognitive theories of psychopathology, each disorder tends to be determined by specific cognitive vulnerabilities, and in our study, three domains were identified as being associated with MD, suggesting the presence of specific dysfunctional beliefs associated with the syndrome. The first domain is *Disconnection/Rejection*: individuals at risk for MD tend to perceive themselves as unlovable, unworthy, and not belonging and others as untrustworthy and rejecting, mistreating, humiliating, and abandoning them. This finding appears to be consistent with the theorizations of Fabris et al. (2018), particularly their assertion that avoidant attachment is strongly linked to MD. Avoidant attachment tends to be associated with relational experiences with one's parents characterised by rejection, emotional distance, and neglect. Individuals with avoidant attachment thus tend to develop a negative image of themselves and their relationships with others, exhibiting an excessive need for self-reliance, discomfort with emotional closeness, and avoidance of intimate relationships, perceived as secondary. Research shows a strong link between the Disconnection/Rejection domain and avoidant attachment (Karatzas et al., 2023). Therefore, MD may be an extreme attempt by individuals to compensate for a negative self-perception as an unlovable subject and to avoid fears of abandonment, rejection, and dependence in interpersonal relationships. The second domain is *Other-directedness*: Adolescents at risk for MD tend to seek admiration and approval from others and to focus more on their own needs than on those of others. Individuals at risk for MD tend to have low self-esteem and may try to invest heavily in their physical appearance in order to secure the approval of others (Martenstyn et al., 2022). In addition, exercise dependence is an important component of MD symptomatology. Haunseblas and Giacobbi (2004) hypothesised that exercise dependence may develop in individuals as a result of irrational beliefs about how improvements in appearance and/or changes in exercise habits may gain the approval of others or meet the expectations of others. Along these lines, some authors (Rankin et al., 2021) found the specific EMS '*seeking approval/recognition seeking'* to be a strong predictor of exercise addiction. The third domain is *Overvigilance/Inhibition*: adolescents at risk for MD tend to have a more pessimistic view of reality, suppress the expression of their feelings, and value adherence to high standards and very strict ethical rules. Schemas pertaining to this domain are more likely to occur in families that are punitive, hypercritical, and prohibit the expression of emotions (Young et al., 2006). This domain is the one most strongly associated with perfectionism (Boone et al., 2013), which in turn is a factor strongly associated with MD (Merhy et al., 2023). In addition, there is evidence that perfectionism mediates the relationship between the *Overvigilante/Inhibition* and body image concerns in youth (Boone et al., 2013). Overall, it is possible that exposure to a family that sets strict rules and high standards and tends to criticise and punish may stimulate the internalisation of these attitudes and promote self-criticism. Perfectionism thus appears to be a way of getting rid of internalised criticism. In this regard, recent findings suggest an association between MD and parental criticism, while other studies (Dryer et al., 2016) find an association between parental pressure, comments, and teasing regarding physical appearance by parents and risk for MD. Therefore, the pursuit of muscularity may be an attempt to achieve high standards of aesthetic perfection in response to internalised critical instances.

Finally, *Impaired Limits* was found to be negatively associated with MD. As outlined above, schemas belonging to the Impaired Limits domain tended to characterise the dysfunctional core beliefs of grandiose narcissism, whereas MD was associated with vulnerable narcissism. In this sense, subjects with high MD scores show a negative and devalued self-perception, so that the pursuit of muscularity could be understood as an attempt to compensate for a self-perception that is perceived as fragile and powerless. However, it is worth noting that theorists seem to agree that, unlike in adulthood, certain EMS might serve an adaptive function in adolescence, and among these, Entitlement/Grandiosity might play a protective role (Nicol et al., 2020).

Finally, regression analyses revealed a relationship between MD and gender. Our data revealed that being male is a significant risk factor for the development of MD. This finding is consistent with the international literature that identifies MD as a typically male disorder (Ganson et al., 2023), probably because musculature is a salient element of male body image and the concept of masculinity tends to be associated with muscularity (Badenes-Ribera et al., 2018). Finally, in terms of gender, we must note that we did not find statistically significant differences in the association between MD and EMS when comparing males and females. This finding may suggest that cognitive vulnerabilities and unmet emotional needs associated with the risk of developing MD may not vary by gender. However, given the exploratory nature of the study, this warrants further investigation, both qualitatively and quantitatively, possibly through recruitment of clinical samples.

## Limits and Future Direction

Our study attempts to fill a gap in the literature by providing preliminary data on the relationship between MD and EMS. However, despite the originality of the study, it is important to consider the limitations of the research. First, although we recruited a large sample of adolescents of both genders, the sample cannot be considered fully representative. Therefore, future studies could investigate the relationship between MD and EMS using a representative sample of adolescents. Second, we used only self-report instruments. This means that subjects' responses may have been influenced by aspects of social desirability, text comprehension, and memory. In addition, self-report instruments cannot substitute for clinical assessment, so MD scores do not necessarily reflect the presence of a clinical diagnosis but rather indicate a level of risk for the syndrome. Future studies may therefore include clinical samples of adolescents with a formal MD diagnosis and/or incorporate multi-informant approaches or alternative assessment methods. Lastly, it is important to note that this was a cross-sectional study, which prevents us from drawing a conclusion about the relationship between MD and EMS in terms of causality. Future studies may therefore use a longitudinal design and enrich the protocols with additional variables of interest (e.g., attachment to parents and peers, perceived parenting style, adverse childhood experiences, etc.) to test complex statistical models and investigate the underlying mechanism.

## Practical Implications

Our study has several practical implications. First, it encourages researchers interested in body image disorders in adolescence to further investigate the possible role of EMS in the development and maintenance of MD. In addition, mental health professionals working with MD patients may benefit from assessing EMS and targeting identified cognitive vulnerabilities in intervention. Finally, the dysfunctional beliefs associated with MD could inform potential interventions to prevent and promote adolescent psychological well-being, particularly in relation to body image and musculature.

## Conclusion

In summary, this is the first study to investigate the relationship between EMS and MD in adolescence, identifying an association between specific EMS (domains) and risk for MD. Male gender emerged as a significant risk factor. Ultimately, our study suggests that EMS represent relevant cognitive vulnerabilities contributing to the development and maintenance of MD symptoms.

## Data Availability

The data that support the findings of this study are available from the corresponding author upon reasonable request.

## Appendix: Early maladaptive Schemas

**Table:**

Domains	Early maladaptive schemas	Brief description
1. Disconnection and Rejection	1. Abandonment/Instability	The belief that others are unstable or not avaible for support and connection and that oneself will be abandoned at any time
	2. Mistrust/Abuse	The belief that others will hurt, humiliate, abuse manipulate or deceive oneself
	3. Emotional Deprivation	The belief that others will not adequately meet one's needs for emotional support
	4. Defectiveness/Shame	The belief that one is flawed, bad, inferior or worthless and that one would be unlovable to others if exposed
	5. Social Isolation/Alienation	The belief that one is isolated from the rest of the world, different from other individuals and/or does not feel one belongs to any group or community
2.Impaired Autonomy and Performance	6. Dependence/Incompetence	The belief that one is unable to take care of oneself when alone
	7. Vulnerability to Harm	The belief that an impending catastrophe will fall on yourself that one will be unable to prevent it
	8. Enmeshment/Undeveloped Self	Excessive emotional involvement and intimacy with one or more significant others (often parents) at the expense of full individuation or normal social development
	9. Failure	The belief that one is not capable or is a failure or is fundamentally inadequate relative to one's peers in areas of achievement
3. Impaired Limits	10. Entitlement/Grandiosity	The belief that one is superior to and better than others, or entitled to special rights and privileges
	11. Insufficient self-control/Discipline	Difficulty in control over one’s impulses and desires and difficulties in tolerating frustration
4. Other Directedness	12. Subjugation	Excessive surrendering of control to others because one feels coerced, usually to avoid anger, retaliation, or abandonment
	13. Self-Sacrifice	Excessive focus on the needs of others in daily situations at the expense of one's own gratification
	14. Approval/Recognition seeking	Excessive emphasis on obtaining approval, attention or recognition from others
5. Overvigilance and Inhibition	15. Negativity/Pessimism	The pervasive focus on negative aspects of life while minimizing or neglecting the positive or optimistic aspects
	16. Emotional Inhibition	Inhibition of emotional expression, usually to avoid disapproval by others, feelings of shame or losingcontrol of one's impulses
	17. Unrelenting Standards/Hypercriticalness	Exaggerated self-criticism and perfectionism, usually to avoid criticism
	18. Punitiveness	The belief that mistakes warrant punishment. Involves the tendency to be angry, intolerant, punitive and impatient with those people (including oneself) who do not meet one's expectations or standards

## Code Availability

For this research we used SPSS 26 sofware available from our University.
